# A Configurable Sensor Network Applied to Ambient Assisted Living

**DOI:** 10.3390/s111110724

**Published:** 2011-11-15

**Authors:** Juan J. Villacorta, María I. Jiménez, Lara del Val, Alberto Izquierdo

**Affiliations:** Departamento de Teoría de la Señal y Comunicaciones e Ingeniería Telemática, Universidad de Valladolid, E.T.S.I. Telecomunicación, Paseo de Belén 15, Valladolid 47011, Spain; E-Mails: marjim@tel.uva.es (M.I.J.); larval@tel.uva.es (L.V.); alberto.izquierdo@tel.uva.es (A.I.)

**Keywords:** Ambient Assisted Living, sensor network, data fusion

## Abstract

The rising older people population has increased the interest in Ambient Assisted Living systems. This article presents a system for monitoring the disabled or older persons developed from an existing surveillance system. The modularity and adaptability characteristics of the system allow an easy adaptation for a different purpose. The proposed system uses a network of sensors capable of motion detection that includes fall warning, identification of persons and a configurable control system which allows its use in different scenarios.

## 1. Introduction

Most of the industrialized countries are trending to an increasingly aging population, causing a rise in the relative percentage of older people in the population. This growing group of older people usually requires greater medical and social attention. Programmes like AAL (Ambient Assisted Living) of the European Commission [1] promote the application of information and communication technologies to enable older or disabled people to remain living independently longer in their own house with an improved quality of life. At the same time, the costs to public health and social systems are reduced by avoiding the need to go to medical or social centers frequently.

The Ambient Assisted Living systems, also called Home Care Systems, are based on the use of a set of sensors interconnected by different types of communication systems in order to get information about the status of the patient and to provide care in his/her own home.

In [2], Ambient Assisted Living solutions are defined as the application of Ambient Intelligence (AmI) technologies to allow that people with specific demands, such as disabled and older persons, adapt their home environment so they can live longer in their own homes. These AmI systems are composed of a top-level control entity which provides intelligence to the system and a network of sensors whose goal is to give the top-level entity answers to W5 + questions [3]:

- Who: identification and tracking of individuals.
- What: recognition of activities and interactions.
- Where and When: providing a space-time framework for the identified actions.
- Why: association of action and identification of tasks and behavior patterns.
- How: track the flow of information through different media.

As AmI systems, security systems have a network of sensors that gather information from the environment to perform the tasks of surveillance and intrusion detection and control access, among other tasks. The difference is in the actions that the top-level module performs from the information obtained. In the case of AmI systems the top-level entity uses that information to improve the users’ quality of life, while the security systems are designed to ensure the integrity of persons and objects monitored. The flexibility of top-level module can transform a system designed for surveillance or AmI into an Ambient Assisted Living system, but after a study of the state of the art we have found no references regarding the adaptation of surveillance or security systems to AAL environments.

This article presents how a multisensor surveillance system [4] can be adapted to the AAL area. The next section presents the system architecture that is detailed in the following two sections describing the high-level entity in the first place and then the implemented sensor network. Section 5 presents two usage scenarios of the proposed system and finally, section 6 shows the conclusions obtained from the work and the future lines of development.

## 2. System Architecture

The designed system presents a modular and distributed structure [5] that can be easily modified to suit the particular conditions of the operating environment and expanded by adding new modules, depending on the specific needs or the future advances in technology.

**Figure 1.**
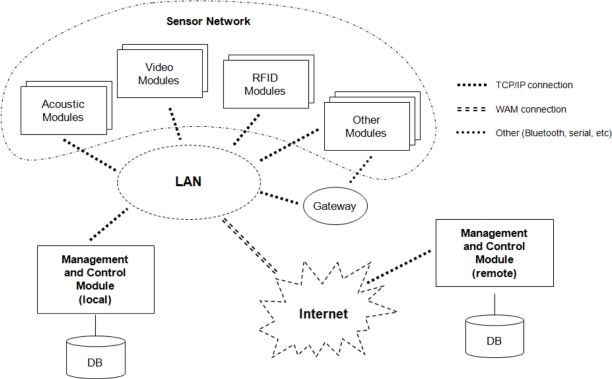
Block diagram of the system.

Figure 1 shows the block diagram associated to the system architecture. The system consists of two types of modules: management and control modules and sensor modules, such as the acoustic module, image module or RFID (Radio-Frequency Identification) module.

The control and management modules are the top-level entity of the system. All implementations must have at least one of these modules which are the central and indispensable element of the system. Its function is to establish communication with the other modules and to manage the tasks performed by each of them. During its operation this module uses a database that stores all information from the system, including its configuration and the data obtained from the various sensor modules.

The sensor modules are used to interact with the environment. Primarily they provide information to the system about what is happening but, in some cases, these modules are also actuators because they can modify the operating environment, e.g., allowing or not a door to open.

The operating environment of the system is divided into areas of activity; each one is characterized by having a set of sensor modules and different operating patterns. This division increases flexibility by allowing the system to perform different behaviors in each area of activity, according to its particular needs.

Communication between the different modules of the system is performed using the TCP/IP through a local area network (LAN). The system can use any data communications infrastructure that supports this protocol, either an existing wired network or a wireless network. However, some of the devices used as sensor modules can be relatively simple and fault to include network capabilities, providing other connection interfaces such as USB or Bluetooth. In order to allow the inclusion of such devices, the system includes a set of gateways or proxies [6] that allow the connection of these devices to the system transparently.

## 3. Management and Control Module

The management and control module, or MCM, is the central and most significant element of the system. It is the one responsible for providing intelligence to the system by controlling the operation of the other modules and for the fusion and management of the information provided by all the elements.

From the hardware point of view, the management and control module comprises a personal computer that is running the different services and applications performed by the module.

### 3.1. Definitions

To perform its functions, the central module uses the concepts of task, event and policy of action [7]:

- A task is the execution of a set of actions by a module or device. These tasks can be of two types: scheduled, if they are programmed to run on a fixed time, or unscheduled, when they are execute after the reception of an event by the system. For each module the system defines the list of tasks that it can perform. The definitions of the tasks are carried out by pro-tasks, which are the abstraction of a task, including the definition of the parameters (number and type) that it will accept when executed.
- An event is the response by the system to an incident during the accomplishment of a task executed by one of the modules. To simplify the operation of the system, the situations of mistake, beginning and ending of a task are also considered to be events. These events are generated by the modules of the sensors network and received by the management and control module, which decides the actions to carry out according to the policies of action.
- The policies of action define the activities to be performed after the arrival of each event. With the arrival of an event, the MCM selects the set of policies associated to it and it decides which policy to apply based on the parameters of the event and the status of the system. The status of the system means the set of events received in an interval of time before the reception of the last event.

### 3.2. Decision Taking and Data Fusion

The Management and Control Module (MCM) implements the intelligence of the system; it receives all the events generated by the sensor network and decides the tasks to perform based on the stored policies of action.

The MCM implements a decision taking motor based on fuzzy logic techniques in which each action policy is proposed as a IF-THEN predicate where the if clause is referred to the arrival of an event with some optional parameters and the then clause is the list of task to perform..

The data fusion is implemented by means of junction operators, like AND or OR. So that, a set of concurrent events from different sensors must arrive in order to fit a policy of action. The time dependence between events can be detailed using time quantifiers, like NEAR or FAR.

This approach gives a lot of flexibility to the system and simplifies the introduction of new policies of action by the system administrator. By default the systems includes a set of predefined policies of action with common interactions between sensors like movement verification between acoustic and video modules.

### 3.3. Management and User Interface

All the information about tasks, events and policies associated with a concrete system implementation is stored in the database connected to the management and control module. Likewise, the system configuration is stored in the database, including the modules that compose the system and their properties. Thus, the individualization and adaptability of the system is based on the information stored in this database. On one hand, the modification of the stored policies of action will influence directly the behaviour of the system, but also the inclusion of new modules in the database and/or the modification of the existing ones will provide new functionalities to the system.

A management application that implements a user interface for accessing and modifying the existing information in the database has been defined. This web application can run directly on the computer that constitutes the management and control module or on any computer connected to the system’s network. There is a user interface that facilitates the introduction and modification of the system data, but it should only be operated by a system administrator with enough knowledge about the operation of the global system and the configuration of the specific implementation, since the modification of the information stored in the database can alter the behavior of the system, making it unstable.

This application includes the following main functionalities:

- Management of environments: it describes the operating environment of the system, including the definition of the areas of activity and the different modules existing in each area.
- Management of tasks: it defines the list of tasks and pro-tasks associated with every module of the system, as well as the different events that each task can generate and the policies assigned to the areas of activity previously defined.
- Management of users: it defines the different authorized users of the system as well as his/her role and his/her levels of access within the system

The system database also stores the status of the system, that is, the information regarding all the events received by the management and control module. The system incorporates an application to consult this information showing both the general information of the system and the historical events received during the time that it has been working.

### 3.4. Local and Remote MCM

In the block diagram of the system shown in Figure 1, there are two different management and control modules: a local module, placed in the same house as the sensor modules and accessible through the local area network, and a remote module, which can be placed anywhere and connected to the system through a wide area network (WAN) such as Internet.

For the proper operation of the system it is only indispensable to have a local module in charge of receiving events and applying the suitable policies. The remote MCM is an optional element and can be viewed as a backup system of the local MCM. The local module sends a copy of the events to the remote MCM, in case that it is enabled in the system implementation. To limit the amount of information sent through the external network link it is possible to select—using the management application—the set of events that are forwarded to the remote MCM.

The remote MCM has a double function: on the one hand, as explained previously, it acts as backup center in which the history of the system is stored. On the other hand, it can act as an alarm center, since the local MCM can invoke a task in the remote MCM as a result of the application of a policy of action. For example, in case that the system detects an accident it can invoke an alarm in the remote system to request the sending of aid to the house where the system is located.

The need to include an alternative method of connection between the local and remote modules is discussed in [6]. This system has not considered this option since the remote module is an optional element that is not essential for the proper functioning of the system and, on the other hand, the local MCM is capable of postponing the sending of the copies of the produced events, if the network link between both control modules is not available.

## 4. Sensor Network

The remaining modules included on the system shown in Figure 1 make up the sensor network used to obtain information from the working environment. The modules implemented in the system are detailed below.

### 4.1. Acoustic Module

This module can be enclosed inside the SODAR (SOund Detection And Ranging) systems that use arrays of sensors to carry out the location and tracking of objects. Although they are based on the philosophy of RADAR systems, these systems differ in that they use acoustic instead of electromagnetic waves [8].

The use of an array of sensors allows, through beamforming techniques [9], to position spatially the monitoring beam by an electronic form. The ability to change the pointing angle quickly and manage several beams at the same time allows these systems to carry out several tasks simultaneously, being able to track multiple objects while detecting the presence of new ones. This is what is known as Multifunction SODAR/RADAR systems or MFAR [10].

Multifunction radars are characterized for including an element called beam manager [11], which is responsible for undertaking the planning of the use of system resources by determining which function is performed in every moment.

The main functions performed by the acoustic module are:

- Surveillance: with the purpose of detecting the presence of new objects that in RADAR terminology are called targets. For this, the system scans the space periodically for new detections.
- Tracking: for every target detected during the vigilance it is necessary to update its position at every time. During the tracking the current position of every target is estimated using Kalman filters [12] and, subsequently, the presence of the object in the estimated position is verified.

In order to implement the functionalities of the acoustic module, specific algorithms of multifunction SODAR systems have been used, taking into account that the operating framework is a closed environment that generates a great quantity of echoes by the reflection of sound waves on the floor, walls and/or other objects. It is necessary to emphasize that in the target detection algorithms, clutter suppression techniques [13] have been applied to minimize false detections.

Taking into account the purpose of the system, we have adapted the target-tracking subsystem to detect, e.g., if a person falls; as this one of the main causes of accidents in the older persons. If a person falls there is an abrupt change of position, from a vertical situation to a horizontal one [14]. The absence of later movement can also be used for the detection of a fall, indicating furthermore that an important damage has taken place. A set of specific detection and tracking algorithms have been implemented to consider this type of changes of position and the consequential generation of falling allarms.

As far as hardware is concerned, the acoustic module is composed by a SBC6713e card made by Innovative Integration that incorporates a DSP TSM320C6713 and a Spartan-II FPGA as processing units; an OMNIBUS card with 16 codecs of 16 bits to perform the acquisition of the signals; a subsystem of signal conditioning formed by 16 pre-amplifiers and signal adapters; and 16 microphones electret in a circular array of 16 elements. It is a stand-alone module that is connected to the whole system using an Ethernet port included in the SBC6713e card.

As far as software is concerned, the FPGA has been used only for the operations of prefiltering and adaptation of the signals obtained by the A/D, while the bulk of the processing (beamforming, detection, task manager, *etc.*) is done by the DSP.

### 4.2. Video Module

The video module is in charge of capturing and processing images from the room in which the module is located. Their integration into the system allows corroborating the information obtained by the acoustic module providing greater reliability to the system.

Video subsystems are traditional elements in security and surveillance systems; however, in the area of Ambient Assisted Living they are not so frequent since they may limit the users’ privacy [6]. Nevertheless when these videos are processed automatically without direct visualization by other people, or its display is only activated in case of emergency [15], privacy is guaranteed.

This module is divided into two subsystems: acquisition and processing. This division allows one to make the module independent from the type of video camera used.

The acquisition subsystem is in charge of controlling the camera itself, resulting in a surveillance image taken from the room under inspection. Since there are different types of cameras, this subsystem has to be tuned for each camera you wish to use. For example, acquisition subsystems have been developed to access and control IP cameras and Webcams connected directly to a PC. In order to achieve independence between the image obtained and the type of camera, we are taken into account, besides the form of access and control of the camera, certain features such as the optical aperture of the lens and the resolution of the obtained images. These configuration data are particularly important in order to merge the information from this module with other modules of the system such as the acoustic module.

The processing subsystem is responsible for managing the sequence of images from the camera in order to carry out the detection of movement. The implemented algorithm uses a variation of the Sakbot algorithm [16], as it provides good results without a high computational load. This algorithm performs the extraction of moving objects by doing a background elimination followed by a segmentation to divide the image into different sections corresponding to the detected objects in movement.

Although the detection of movement with this technique is trustworthy enough, a small drawback is to transfer the position of the object in the two-dimensional image to a three-dimensional position in a closed space. The module can take into account the optical characteristics of the camera, the position and relative dimensions of the detected object to predict the position of the object, but this approach is not very reliable. For this reason, the purpose of the process is the confirmation of the detections made with the acoustic module following three scenarios:

- Verification of acoustic detection: after a change in the position of a detected target by the acoustic module, it verifies whether there has been a detection of movement in the video module for the corresponding position.
- Motion detection: a detection of movement associated to an object in the image sequence invokes the verification through the acoustic module, in order to obtain a reliable position.
- Fall detection: using an algorithm for extracting vertical-horizontal dimensions of the detected objects [17], it generates a fall detection event when a sudden change is detected. This event can be verified with information provided by the acoustic module.

The hardware part of this module is composed by the camera to capture images and a personal computer running both acquisition and processing subsystems. This computer can be the same in which the MCM is implemented or a different one, for example, an embedded computer specifically dedicated to these tasks.

### 4.3. RFID Module

Radio-Frequency Identification technology or RFID [18] allows the secure identification of electronic devices wirelessly, without any physical contact between these devices. Passive RFID tags are small items that do not require batteries to operate, so they can be placed in everyday life objects. These RFID tags are used together with RFID readers that can detect and identify the proximity of such devices.

There are AAL’s systems that use devices RFID to help the patient locate lost objects: the placement of RFID tags in a variety of objects allows their location, if there is a network of RFID readers distributed in the house [3] or if the patient carries a small portable reader [19]. The use of RFID tags placed in the clothes or a bracelet in order to determine the patient position by means of a RFID network has also been documented [19].

Since the system has available methods to locate the patient within the house, the purpose of the module implemented in the proposed system is purely for identification and access control. Each person who usually enters the house (patient, family, health personnel, *etc.*) is given a bracelet that includes a passive RFID device. RFID readers are distributed and placed on the doors of the rooms. When a person moves from one room to another, the RFID reader on the door detects the bracelet and identifies the person wearing it. This simple module is supplementary to the acoustic and video modules, since it allows assigning an identity to objects/targets detected by the other modules, allowing determining exactly where each person is placed at any given moment.

Besides its identification functionality, each RFID reader controls the operation of the electronic lock of the door, allowing or not the access to the room depending on the identity of the person that is going to touch it. As mentioned above, the main function of this module is only to identify the person accessing the room, not to deny the access to certain areas of the house. Nevertheless, in case of people with diseases characterized by episodes of disorientation such as Alzheimer’s the RFID reader can also be used to prevent the subject from accessing to dangerous areas or from leaving the house. Or in less serious cases, to trigger an alarm when the patient is going into a dangerous zone.

The hardware implementation of this module has been designed as a made-to-measure system composed of an electronic key, an RFID reader with serial interface and a serial to Ethernet converter.

### 4.4. Other Modules

The flexibility of the system architecture allows the addition of different devices depending on the needs of each particular case. The only requirement is that the device should incorporate a network interface. Nevertheless, as explained previously, this is not a critical problem because the system provides procedures for the creation of gateways to transform any other communication interface to the one required by the system.

Some examples of other modules included in the system are:

- Panic button: a large button positioned in an easily accessible place. When pressed, it indicates that an emergency has occurred and that medical or social services should be alerted. This module is implemented using a circuit specifically designed for this task, using an Atmel ATmega128 microcontroller and a module that incorporates an Ethernet interface.
- GSM module: it triggers an alert in case of an emergency by sending an SMS to a telephone number previously stored in the system, e.g., a family member or an emergency medical service. It is also possible to send a multimedia message with a capture by the video module. This module can be implemented using any standard GSM cellular phone with Bluetooth interface. This interface is used to connect thought PC to the MCM using a Bluetooth to TCP-IP gateway.
- Display module for notices: it notifies important information to the patient or caregiver. For example, a reminder to the patient to take his/her medication or an alert to the nurse of an emergency has occurred. It uses a standard screen connected to a computer that could be the one used for the MCM or another one in the house. Also, a digital television can be used, as many of them have a serial interface for control and configuration, which would allow power-on of the television and commutation to the correct channel before information from the AAL system is shown.
- Module for measuring vital signs: there are different portable devices on the market that allow the measurement of people's vital signs such as temperature, heart rate, *etc.* This information is sent to the management and control module through a Bluetooth connection so that the system can track the patient's condition and activate an alarm when there is a dangerous alteration of the vital signs.

## 5. Study Cases

The developed system can be applied to very different and complex scenarios. This section presents two concrete examples of application of the system.

### 5.1. Nursing Home

This case study is applicable to disabled or older persons who have a live-in nurse to assist them at home. It also applies to residential medical complexes in which every patient has a set of rooms for personal use (studio or mini-apartment) with a set of common areas and medical staff in a nearby place for if emergencies arise.

The purpose of the system in this case is to provide greater privacy and independence to the patient, since it is not necessary the presence of the nurse all the time. In case of emergency, the system will alert the nurse that will come immediately.

**Figure 2.**
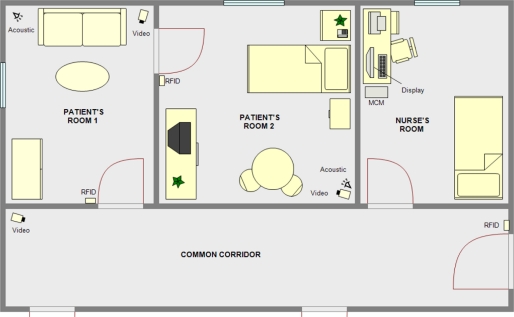
Example scenario of nursing home case of study.

Figure 2 shows an example of a home scenario. The patient has two interconnected rooms for his/her own use. Each room is equipped with an acoustic module and a video module. On the access doors to each room, there is an RFID module for identification and access control. The rooms of the patient and the nursés are connected by a corridor where a video module has been placed. Finally, in the room assigned to the nurse there is a computer that incorporates the MCM and a display module.

In the case of a medicalized disabled/old people’s home, the distribution would be similar, except with more private bedroom areas with access along the same corridor. Figure 2 shows the doors that communicate these additional private areas along the corridor.

Action policies can be adjusted depending on the needs of the patient. For example:

- A patient due to his/her deficiencies or diseases should remain lying down. Such a patient should, therefore, stay in bed all the time. When the patient is alone, there should be no movement in either of the two rooms. Motion detection by the acoustic module and verified by the video module will generate an alarm on the monitor of the nurse. In case any authorized person (nurse, family, *etc.*) access the patient’s area, the monitoring system will be disabled until that person leaves the room.
- A patient with episodes of disorientation. The patient movement’s within his/her private rooms is allowed, so the detection of movement does not generate an alert to the nurse’s monitor. Depending on the severity of the disease, the patient is allowed or not, to go out of his/her private area to the common corridor. In any case, leaving the private area will generate a warning on the display module of the nurse.
- A patient in relatively good health. This is the simplest case. The patient can move without restrictions around his/her rooms and even go out to the common area. The system will record his/her movements and in case of an accident, such as a fall, takes place, it fires an alert.

### 5.2. Autonomous System at Home

In this possible scenario the disabled/older person is living alone at his/her own home. The proposed system is responsible for providing the user both independence and security, knowing that, in case of accident he/she is being monitored and will be assisted immediately.

**Figure 3.**
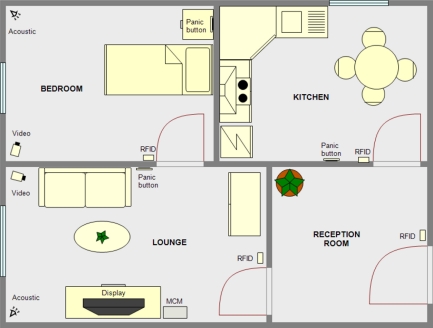
Example scenario of autonomous system at home case of study.

Figure 3 shows a possible distribution of housing for this scenario. The house has four rooms. In the bedroom and lounge, the two most used rooms, both an acoustic and a video one have been installed in order to track people’s movements and detect possible falls.

All the doors have RFID modules to identify the inhabitants and easily determine in which room the patient is, as the access of a person (identified or not) to any room is never denied. Finally, the management and control module has been placed in the lounge where a digital television is also used as display module to show the notices from the system. The last elements of the system are panic buttons, distributed in places of easy access, and a GSM module.

This scenario is a case of minimal interference for the user, as the system remains asleep and it only starts operating under certain circumstances:

- When an emergency takes place, for example when a fall is detected or when any of the panic buttons is pressed, the system sends a text message to a pre-programmed telephone number.
- The system stores information about when the user has to take his/her medication and activates the digital television via the serial interface incorporated and displays a reminder with the medicine and dosage to take.

This scenario is suitable for the use of remote management and control module, which could be located in a medical or welfare center to which the patient is subscribed. In case of emergency, the remote module would be activated.

### 5.3. Testing and Results

To prove the validity of the developed system prototypes of both proposed scenarios were built. In these scenarios we carried out multiple tests simulating real live situations with different actors as users.

In the first tests we have only included in the MCM the predefined set of action policies, that includes the policies associated the with the default behavior or the modules and the sensor data fusion policies: for example between the video and acoustic motion detection or assigning a identity to a moving object through a RFID module. With these test we have verified the correct functioning of the system. Although detection and tracking of moving objects by the video and acoustic modules had already been verified with the surveillance systems used previously [4]. In these tests, the correct functioning of the modifications introduced for the detection of falls and the usefulness of the double video-acoustic check to reduce the number of false alarms was validated.

We also verified the correct functioning of the new modules like the RFID ones. Regarding the RFID module, we have detected a certain rate of uncertainty in the identification of the persons under certain and very specific circumstances. For example, when there are two people in the same room close together and without moving, the acoustic module is unable to determine the identity of each one when they begin to move. Therefore, in this situation, the system may confuse the identity of people in movement until he/she approaches to a RFID module that performs a new identification. The solution to this problem is to add more RFID readers in the room.

We also found that the accuracy in the monitoring of movement can allow us to identify symptoms of disorientation by detecting the erratic movements of the patient. Specific algorithms should be developed in order to automate the detections of these events.

In the next stage of the tests we introduced the specific action policies associated with each case study. The existence of the default set of action policies reduces the number of policies to add and simplifies the configuration process. In the first case study the action policies to include are different depending of the state of the patient but we only have to modify two or three policies to go from one case to another. The second case study is simpler to implement because most of the default action policies fit the scenario and we only have to configure some settings of these policies, like the telephone number of the GSM module or the scheduler for the activation of the display module. In both cases we have prove that the system can be adapted to different situations easily. The tests carried out in the different scenarios with the new action policies validate that the system works fine in each case and that no more action policies should be added to fit the study case.

## 4. Conclusions

This article presents a system for monitoring older or disabled people developed from an existing surveillance system. The main keys of the presented system are:

- The system includes different types of sensors for the detection, identification and tracking of people in the workspace.
- The use of an innovative acoustic sensor which uses beamforming techniques and its interaction with the video module.
- The system includes a management and control module that, through the use of action policies, is responsible for merging the information from all existing modules and making suitable decisions.
- The system can be adapted to different situations as shown in the explained case studies. The use of action policies allows the modification and adaptation of the system behaviour to each particular case.
- We have tested the system in several real environments, using actors as users, validating the adaptability through simple modifications on the action policies defined in the system.

After successful testing of the system in simulated environments, we are in talks with some local geriatric centers to incorporate a prototype in the facilities so we can try the system in real scenarios. At this time the work team is focused on several lines of development, including the incorporation of new modules or the identification of episodes of disorientation. Likewise, we have experimented with acoustic signature techniques to identify people in order to assign an identity to the targets detected by the acoustic module. The obtained results with the acoustic signature, although promising, are still too preliminary for their inclusion in the developed system presented in this paper.
